# Antiangiogenesis-Combined Photothermal Therapy in the Second Near-Infrared Window at Laser Powers Below the Skin Tolerance Threshold

**DOI:** 10.1007/s40820-019-0327-4

**Published:** 2019-10-31

**Authors:** Jian-Li Chen, Han Zhang, Xue-Qin Huang, Hong-Ye Wan, Jie Li, Xing-Xing Fan, Kathy Qian Luo, Jinhua Wang, Xiao-Ming Zhu, Jianfang Wang

**Affiliations:** 1 State Key Laboratory of Quality Research in Chinese Medicine, Macau Institute for Applied Research in Medicine and Health, Macau University of Science and Technology, Taipa, Macau SAR People’s Republic of China; 2 Department of Physics, The Chinese University of Hong Kong, Shatin, Hong Kong SAR People’s Republic of China; 3 Faculty of Health Sciences, University of Macau, Taipa, Macau SAR People’s Republic of China; 4 Beijing Key Laboratory of Drug Targets Research and New Drug Screening, Institute of Materia Medica, Chinese Academy of Medical Sciences and Peking Union Medical College, Beijing, 100050 People’s Republic of China

**Keywords:** Antiangiogenesis therapy, Core@shell nanostructures, Gold nanobipyramids, Photothermal therapy, Plasmon resonance

## Abstract

Photothermal agents with strong light absorption in the second near-infrared (NIR-II) region (1000–1350 nm) are strongly desired for successful photothermal therapy (PTT). In this work, titania-coated Au nanobipyramids (NBP@TiO_2_) with a strong plasmon resonance in the NIR-II window were synthesized. The NBP@TiO_2_ nanostructures have a high photothermal conversion efficiency of (93.3 ± 5.2)% under 1064-nm laser irradiation. They are also capable for loading an anticancer drug combretastatin A-4 phosphate (CA4P). In vitro PTT studies reveal that 1064-nm laser irradiation can efficiently ablate human lung cancer A549 cells and enhance the anticancer effect of CA4P. Moreover, the CA4P-loaded NBP@TiO_2_ nanostructures combined with PTT induce a synergistic antiangiogenesis effect. In vivo studies show that such CA4P-loaded NBP@TiO_2_ nanostructures under mild 1064-nm laser irradiation at an optical power density of 0.4 W cm^−2^, which is lower than the skin tolerance threshold value, exhibit a superior antitumor effect. This work presents not only the development of the NBP@TiO_2_ nanostructures as a novel photothermal agent responsive in the NIR-II window but also a unique combined chemo-photothermal therapy strategy for cancer therapy.

**Electronic supplementary material:**

The online version of this article (10.1007/s40820-019-0327-4) contains supplementary material, which is available to authorized users.

## Highlights

- Titania-coated Au nanobipyramids (NBP@TiO_2_) with a high photothermal conversion efficiency in the NIR-II window are synthesized.
- The TiO_2_ shell is capable for the loading of the anticancer drug combretastatin A-4 phosphate (CA4P).
- The CA4P-loaded NBP@TiO_2_ nanostructures subjected with photothermal therapy induce synergistic anticancer and antiangiogenesis effects under 1064-nm laser irradiation at a power density lower than the skin tolerance threshold value.

**Electronic supplementary material:**

The online version of this article (10.1007/s40820-019-0327-4) contains supplementary material, which is available to authorized users.

## Graphical abstract

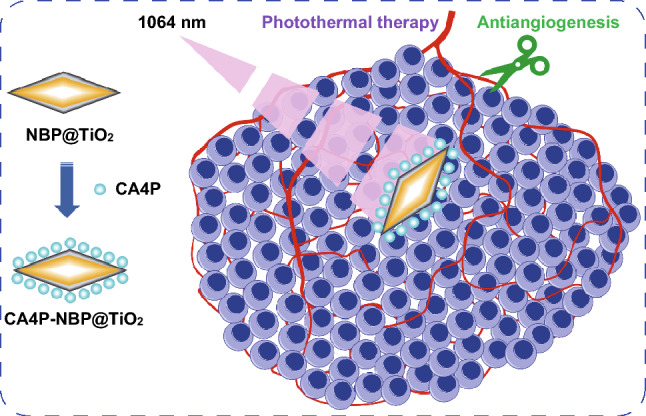

## Introduction

Photothermal therapy (PTT) using near-infrared (NIR) laser irradiation is an emerging treatment for cancer therapy [1]. This approach involves the conversion of light into heat to cause damage to cancer cells. Compared with conventional cancer therapies, this method has the advantages of minimal invasiveness, high temporal and spatial control, and fewer side effects owing to the absence of toxicity for the light-absorbing agents in dark. The NIR (650–950 nm, NIR-I) light is commonly used for PTT. Recently, light in the second NIR (NIR-II) window (1000–1350 nm) has been suggested to provide a much deeper tissue penetration due to less light absorption and scattering by tissues and blood [2, 3]. The penetration depth is deemed to be maximal for light in the wavelength range of 1000–1100 nm [4]. In addition, maximal permissible exposure for skin is higher for light in the NIR-II window. According to the skin tolerance threshold set by the American National Standards Institute, a 1064-nm laser has a higher maximal permissible exposure (1.0 W cm^−2^) than that of an 808-nm one (0.33 W cm^−2^) [5].

There has recently been rapidly growing interest in the development of photothermal agents responsive in the NIR-II window. Multi-branched Au nanoechinus [6], Au–Cu_9_S_5_ [5], Au nanorod-in-shell [7], black hollow silica [8] (NH_4_)_*x*_WO_3_ [9], donor–acceptor structured [10, 11], and semiconducting [12, 13] polymer nanoparticles have been reported to be excitable under 1064-nm (NIR-II) laser irradiation and cause photothermal ablation of cancer cells. However, most of these photothermal agents show a very broad absorption in the NIR-II region and in general have low photothermal conversion efficiencies (< 50%) [14, 15]. In order to achieve a good PTT efficacy, laser irradiation at high optical powers, even far beyond the skin tolerance threshold value, has been used [7], which is not applicable for in vivo therapy. Therefore, the search for photothermal agents with higher photothermal conversion efficiencies in the NIR-II window has remained to be highly desirable.

Localized surface plasmon resonance (LSPR) is an intrinsic property of noble metal nanocrystals. It enables noble metal nanocrystals to function as photothermal agents [16]. Plasmonic nanostructures that support LSPRs are the most widely studied, and encouraging progress has been achieved for PTT. Among them, Au nanostructures, such as Au nanorods [17], Au nanocages [18], and Au nanoshells [19], show synthetically controllable LSPR properties, which can be tailored through the control of their shape, size, and aspect ratio [20]. However, their absorption bands lie mostly in the NIR-I window.

The best PTT efficacy of a plasmonic photothermal agent is achieved when its plasmon resonance wavelength matches the wavelength of laser irradiation [21]. Till now, the construction of Au nanostructures with LSPR bands extending above 1000 nm has remained rare. We have recently synthesized Au nanobipyramids (NBPs) in high purity. Their LSPR can be tailored to the spectral region covering the entire NIR-II window [22]. Compared with Au nanorods, which are also elongated in one direction, Au NBPs are highly uniform in shape and size. Due to the penta-twinned crystalline structure, each Au NBP has two sharp ends, which endow Au NBPs with much larger local electromagnetic field enhancements than Au nanorods. As a result, Au NBPs have smaller plasmon linewidths, including both homogeneous and inhomogeneous ones, and higher refractive index sensitivities than Au nanorods. However, the PTT application of Au NBPs has remained relatively unexplored.

On the other hand, with increasing demand of thorough ablation of tumors, rigorous photothermal heating to high temperatures over 50 °C is required to induce complete cell necrosis. But high hyperthermia temperatures induced by strong laser irradiation can inevitably damage the normal tissues nearby a tumor owing to the nonspecific heat diffusion [23]. This greatly limits the further clinical applications of PTT. Though mild hyperthermia therapy (e.g., 45 °C) is acceptable, it is insufficient to fully ablate tumors. Recent studies have shown that a combination of mild PTT with other types of therapies, such as chemotherapy, using multifunctional platforms can further improve the outcomes of cancer therapy [24, 25].

Besides chemotherapy, antiangiogenesis therapy has also been accepted to starve tumors by blocking their blood supply [26]. Combretastatin-A4 phosphate (CA4P), structurally similar to colchicine, binds tubulin at the colchicine binding site and can potently disrupt the polymerization of tubulin cytoskeleton [27]. It can inhibit tumor cell proliferation by arresting mitosis through tubulin binding. In addition, the disruption of tubulin by CA4P can also result in changes in the tumor endothelial shape and therefore rapidly shut down the blood flow around the tumor. CA4P-based antiangiogenesis therapy has been in clinical development for treating ovarian and other cancers. The combination of CA4P with other therapies is expected to give substantially improved outcomes in cancer therapy. When hyperthermia therapy and CA4P are combined, a synergistic therapeutic benefit can take place [28].

In this study, titania-coated Au NBPs (NBP@TiO_2_) are employed as a multifunctional nanoplatform for both PTT in the NIR-II window and the delivery of CA4P. The synthesized NBP@TiO_2_ nanostructures have a longitudinal plasmon resonance wavelength of 1064 nm, which enables them to function as an attractive photothermal agent in the NIR-II window. The nanostructure sample shows a high photothermal conversion efficiency under 1064-nm laser irradiation and displays good biocompatibility. In addition, the TiO_2_ coating functions as an effective carrier for CA4P. The obtained CA4P-loaded NBP@TiO_2_ nanostructures enable the simultaneous delivery of CA4P and heat. The enhanced anticancer and antiangiogenesis activities of the combined chemo-photothermal therapy are observed in human lung cancer A549 cells and human umbilical vein endothelial cells (HUVECs), respectively. In vivo studies in A549 tumor-bearing nude mice verify the in vitro results. The combined therapy shows a superior antitumor effect on the inhibition of the micro-vessel density and cancer cell proliferation. Our work presents a new strategy for chemo-photothermal therapy using the NBP@TiO_2_ nanostructures, which are responsive in the NIR-II window. The results will be of importance for the future clinical translation of this optical cancer therapy method.

## Experimental Section

### Growth of the Au NBPs

A seed-mediated growth method [22] was used to prepare the Au NBPs. Briefly, the citrate-stabilized seed solution was prepared by adding a freshly prepared, ice-cold NaBH_4_ solution (0.01 M, 0.15 mL) into a mixture solution made of HAuCl_4_ (0.01 M, 0.125 mL), trisodium citrate (0.01 M, 0.25 mL), and water (9.625 mL). The obtained seed solution was kept at room temperature for 2 h before use. The growth solution was prepared by the sequential addition of HAuCl_4_ (0.01 M, 1.2 mL), AgNO_3_ (0.01 M, 0.6 mL), and ascorbic acid (0.1 M, 0.4 mL) into an aqueous cetyltributylammonium bromide (CTBAB) solution (0.01 M, 28.5 mL). After gentle inversion for 10 s, the seed solution (0.2 mL) was rapidly added into the growth solution. The resultant solution was mixed by stirring for 30 s and then kept at 30 °C overnight.

The as-grown NBP sample (40 mL) was centrifuged at 5000 rpm for 10 min, and the obtained pellet was redispersed in an aqueous cetyltrimethylammonium chloride (CTAC) solution (0.08 M, 30 mL), followed by the subsequent addition of AgNO_3_ (0.01 M, 8 mL) and ascorbic acid (0.1 M, 4 mL). The reaction solution was then kept at 60 °C for 4 h. After centrifugation (3500 rpm, 10 min), the obtained precipitate was redispersed in a cetyltrimethylammonium bromide (CTAB) solution (0.04 M, 20 mL) and left undisturbed at room temperature overnight. The supernatant was discarded, and the remaining precipitate was redispersed in water (10 mL). CTAB solution (0.1 M, 0.2 mL), NH_3_·H_2_O (30 wt%, 0.8 mL), and H_2_O_2_ (0.1 M, 0.6 mL) were subsequently added. The resultant solution was kept at room temperature overnight. The clear supernatant was then centrifuged at 5000 rpm for 10 min. The obtained precipitate was redispersed in water (20 mL) for further use.

### Preparation of the NBP@TiO_2_ Nanostructures

The NBP@TiO_2_ nanostructures were prepared according to a previous work [29]. The NBPs were first coated with poly(sodium 4-styrenesulfonate) (PSS). Briefly, the above Au NBP solution (20 mL) was added dropwise into a PSS solution (molecular weight: 70,000, 2 g L^−1^, 20 mL, 6 × 10^−3^ M NaCl), followed by stirring for 6 h. The excess PSS molecules were removed by centrifugation (5000 rpm, 10 min), and the resultant pellet was redispersed into water (0.4 mL). The TiO_2_ precursor solution was separately prepared by adding a TiCl_3_ solution (15 wt%, containing 20–30 wt% HCl, 0.4 mL) into water (12 mL), followed by the dropwise addition of a NaHCO_3_ solution (0.93 M, 1.9 mL) under stirring. The above concentrated PSS-coated NBP sample (0.4 mL) was then added immediately into the TiO_2_ precursor solution. After being stirred for 30 min, the produced NBP@TiO_2_ nanostructures were precipitated by centrifugation (5000 rpm, 10 min) and redispersed in water (10 mL). For the in vivo studies, the NBP@TiO_2_ nanostructures were further functionalized with poly(ethylene glycol) (PEG). Briefly, the NBP@TiO_2_ nanostructure solution (1200 μg-Au mL^−1^, 100 μL) was mixed with a methoxy-PEG-dopamine solution (molecular weight: 5000, ToYongBio, Shanghai, China, 5 mg mL^−1^, 100 μL) and stirred for 2 h. The PEG-coated NBP@TiO_2_ nanostructures were then collected by centrifugation (5000 rpm, 10 min).

### Characterization of the NBP@TiO_2_ Nanostructures

The transmission electron microscopy (TEM) images of the NBP@TiO_2_ nanostructures were captured on an FEI Tecnai Spirit microscope operated at 120 kV. High-angle annular dark-field scanning transmission electron microscopy (HAADF-STEM) characterization and elemental mapping were performed on an FEI Titan G2 60-300 microscope. The extinction spectra were measured on a Lambda 950 ultraviolet/visible/NIR spectrophotometer. The Au mass concentrations in the solution samples were measured on an Agilent 7500a inductively coupled plasma atomic emission spectrometry (ICP-AES) system. Fourier transform infrared spectroscopy (FTIR) analysis was performed on a Shimadzu IRAffinity-1S spectrophotometer. Thermogravimetric analysis (TGA) was carried out on a Perkin Elmer TGA 6 thermogravimetric analyzer, using a heating rate of 1 °C min^−1^. The hydrodynamic size of the CA4P-loaded NBP@TiO_2_ nanostructures was measured by a Malvern Zetasizer Nano ZS90 size analyzer.

### Determination of the Photothermal Conversion Efficiency

An aqueous NBP@TiO_2_ nanostructure solution (2 mL) in a 1-cm square cuvette, with the optical density at 1064 nm adjusted to 2.0, was irradiated with a 1064-nm laser (Changchun New Industries Optoelectronics Tech. Co., Ltd., China) at different powers (0.4, 0.6, 0.8, and 1.0 W) for 20 min. The temperature of the solution was recorded with a thermocouple microprobe. The probe was completely submerged in the solution without direct exposure to the laser light. The cooling curve was measured after the laser was switched off. The photothermal conversion efficiency of the NBP@TiO_2_ nanostructures was calculated according to a method developed by us previously [21].

### CA4P Loading

The NBP@TiO_2_ nanostructure solution (1200 μg-Au mL^−1^, 100 μL) was centrifuged at 5000 rpm for 10 min, and the obtained precipitate was redispersed in a CA4P solution (350 μM, 100 μL, Selleck Chemicals, Houston, TX, USA) by gentle ultrasonication for 10 s. The mixture was stirred overnight at room temperature and then centrifuged at 5000 rpm for 10 min. The supernatant was subsequently collected, and the concentration of CA4P in the supernatant solution was analyzed on an Agilent 6230 time-of-flight liquid chromatography–mass spectrometry (LC–MS) system. The drug encapsulation efficiency was calculated as the percentage of the loaded drug amount relative to the total drug amount, where the loaded drug amount was taken as the difference between the total drug amount and the amount of the drug in the supernatant.

### Drug Desorption Assay

The above-obtained CA4P-loaded NBP@TiO_2_ nanostructure solution (100 μL, 120 μg Au) was added into 900 μL of water, phosphate solutions (2 and 12 mM PO_4_^3−^), and a citrate buffer (20 mM, pH 4.5). The mixed solution was stirred at 37 °C for 0–48 h. At each time point, a portion (60 μL) of the solution was taken out and water (60 μL) or the fresh buffer was supplemented for further incubation. The collected solution was centrifuged at 12,000 rpm for 10 min, the CA4P concentration in the supernatant was measured by LC–MS, and the relative drug release percentage was calculated.

### Cell Culture

Human lung adenocarcinoma A549 cells were obtained from American Type Culture Collection (ATCC, Manassas, VA, USA) and cultured in Roswell Park Memorial Institute 1640 (Thermo Fisher Scientific, Waltham, MA, USA) containing 10% fetal bovine serum, 100 U mL^‒1^ penicillin, and 100 μg mL^‒1^ streptomycin. HUVECs were obtained from Angiocrine Bioscience (New York, NY, USA) and cultured in an EBM-2 endothelial cell growth medium BulletKit (Lonza, Walkersville, MD, USA). Only HUVECs at a passage of 4–8 were used.

### Cell Viability Assay

The viability of cells was determined using the 3-(4,5-dimethyl-2-thiazolyl)-2,5-diphenyl-2-H-tetrazolium bromide (MTT) assay. 10,000 HUVECs or 6000 A549 cells were seeded into each well on a 96-well plate. After incubation for 24 h, the medium was replaced with the fresh medium containing the NBP@TiO_2_ nanostructures at different concentrations. After 48-h treatment, the medium was discarded, and a fresh medium (100 μL) containing MTT (0.5 mg mL^−1^) was added into each well. After incubation for 3 h, the medium was discarded and the resultant formazan crystal was dissolved with dimethyl sulfoxide (150 μL). The absorbance of each well was measured using a SpectraMax Paradigm multimode microplate reader (Molecular Devices, Sunnyvale, CA, USA) at 540 nm. The cell viability for each sample relative to control was calculated.

Calcein acetoxymethyl ester (calcein AM) staining was also used to determine the cell viability. After the treatment, the medium was replaced with a fresh medium containing calcein AM (2 μM, Thermo Fisher Scientific). After incubation for 30 min at 37 °C, the cells were washed with a fresh medium. The green fluorescence of the cells was observed under an Olympus IX71 fluorescence microscope.

### Tube Formation Assay

Phenol red-free Matrigel (8.5 mg mL^−1^, 100 μL, Corning, NY, USA) was added into each well on a 48-well plate. After incubation at 37 °C for 1 h, 25,000 HUVECs suspended in the medium (200 μL) were seeded on the top of Matrigel. After further incubation for 1 h, the medium was changed with a fresh medium containing the NBP@TiO_2_ nanostructures (12 μg-Au mL^−1^, 250 μL) or the CA4P-loaded NBP@TiO_2_ nanostructures (7 nM CA4P, 12 μg-Au mL^−1^, 250 μL), and PTT was conducted. The cells were finally stained with calcein AM after further incubation for 12 h, and the tubule structure was visualized on a fluorescence microscope. The tube formation was also quantified by calculating the lengths and areas of the tubule structures using an Image-Pro Plus 6.0 software.

### PTT

A total of 6000 A549 cells were seeded into each well on a 96-well plate. After incubation for 24 h, the cells were treated with the NBP@TiO_2_ nanostructures (100 μg-Au mL^−1^, 100 μL), followed by further incubation for 24 h. For the PTT study, the cover of the plate was removed, and the desired wells were exposed to 1064-nm laser irradiation for 5 min. The laser power densities were set in the range of 0–0.9 W cm^−2^. After the cells were incubated for 24 h, the MTT assay and calcein AM staining were performed separately to determine the cell viability.

For the PTT study in HUVECs, the cells were treated with the NBP@TiO_2_ nanostructures (12 μg-Au mL^−1^, 100 μL). After incubation for 24 h, the cells were exposed to 1064-nm laser irradiation at the power density in the range of 0–2.3 W cm^−2^ for 3 min.

### Combination of PTT and CA4P Treatment

A total of 6000 A549 cells were seeded into each well on a 96-well plate. After incubation for 24 h, the cells were treated with the CA4P-loaded NBP@TiO_2_ nanostructures (15 nM CA4P, 100 μg-Au mL^−1^), followed by further incubation for 24 h. The cells were then exposed to 1064-nm laser irradiation for 5 min. For the combined therapy in HUVECs, the HUVECs were treated with the NBP@TiO_2_ nanostructures (7 nM CA4P, 12 μg-Au mL^−1^) for 24 h and then exposed to 1064-nm laser irradiation at 1.8 W cm^−2^ for 3 min. After 24 h, the MTT assay and calcein AM staining were performed separately to determine the cell viability.

### β-Tubulin Immunofluorescence Assay

A total of 10,000 HUVEC cells were seeded in each well of a 96-well plate. After incubation for 24 h, the cells were treated with the NBP@TiO_2_ (12 μg-Au mL^−1^) or the CA4P-loaded NBP@TiO_2_ nanostructures (3 nM CA4P, 12 μg-Au mL^−1^) for 24 h, followed by 1064-nm laser irradiation (1.3 W cm^−2^, 3 min). After further incubation for 24 h, the cells were washed with phosphate-buffered saline (PBS), fixed with 4% paraformaldehyde for 15 min, and then permeated with 0.1% Triton X-100 for 15 min. The cells were subsequently stained with Hoechst 33342 (100 ng mL^−1^, Thermo Fisher Scientific) for 40 min. After being washed with PBS, the cells were further incubated with a rabbit anti-β-tubulin primary antibody (#2146, 1:200, Cell Signaling Technology, Beverly, MA, USA) for 2 h, followed by incubation with an Alexa Fluor 555-conjugated goat anti-rabbit second antibody (1:1000, Thermo Fisher Scientific) for 2 h. The fluorescence images of the cells were finally captured on a fluorescence microscope.

### Xenograft Assay

(6–8 week)-old female BALB/c nude mice were subcutaneously injected with 1 × 10^7^ A549 cells suspended in PBS (100 μL) at hind limbs. The tumor sizes were monitored every 2 days using a caliper. The volumes were calculated according to the equation of volume = (tumor length) × (tumor width)^2^/2. When the tumor growth reached approximately 80 mm^3^, the mice were subjected to further treatments. All procedures were carried out in accordance with Animal Inspection and Control of Macau and were approved by the Animal Care and Use Committee of Macau University of Science and Technology.

### In Vivo CT Imaging

The tumor-bearing mice were intratumorally injected with 25 μL of the PEG-coated NBP@TiO_2_ nanostructures (25 mg-Au kg^−1^ body weight). The mice were then anesthetized by intraperitoneal injection of pentobarbital sodium (1.5 wt%, 100 μL) at a dosage of 75 mg kg^−1^ body weight. The computed tomography (CT) images were acquired by a Bruker SkyScan 1176 in vivo micro-CT scanner (Kontich, Belgium).

### In Vivo PTT

A549 tumor-bearing mice were randomly divided into six groups for different treatments as follows: Group I, control group without any treatment; Group II, laser irradiation only; Group III, NBP@TiO_2_ nanostructure injection only; Group IV, PPT group with the NBP@TiO_2_ nanostructure injection together with the laser irradiation; Group V, chemotherapy group with the CA4P-loaded NBP@TiO_2_ nanostructure injection only; and Group VI, combined treatment group with the CA4P-loaded NBP@TiO_2_ nanostructure injection together with the laser irradiation. The NBP@TiO_2_ nanostructures with or without CA4P loading were intratumorally injected at the dose of 25 mg kg^−1^ in terms of Au, while the injection dose of CA4P was 2 mg kg^−1^. The mice received corresponding injection at day 0. After 24 h (day 1), the mice of Group II, IV, and VI were irradiated with the 1064-nm laser at the power density of 0.4 W cm^−2^ for 5 min. The thermographic photos of the mice were acquired using a FLIR One Pro thermal camera (FLIR Systems, Inc., Portland, OR, USA) during the laser irradiation. Since the beginning of the treatment (day 0), the tumor sizes were measured using a caliper every 2 days. All mice were killed on day 20, and the tumor tissues were collected for the immunofluorescence analysis.

### Analysis of the Tumor Vessel Density and Ki67 Expression

The tumors were fixed with formalin solution (10%) for 12 h and stored at − 80 °C. Frozen sections with a thickness of 10 µm were then prepared using a Leica CM3050S cryostat (Leica Biosystems, Nussloch GmbH, Nussloch, Germany) and washed with tris-buffered saline (TBS). After permeabilization (0.1% Triton X-100 in TBS, TBST) for 15 min and blocking (5% bovine serum albumin in TBST), the primary antibody rabbit anti-CD31 (1:200, ab28364, Abcam, Cambridge, UK) or rabbit anti-Ki67 (1:200, ab15580, Abcam) was applied overnight at 4 °C, followed by incubation with Alexa Fluor 488- or Alexa Fluor 555-conjugated goat anti-rabbit secondary antibody (Thermo Fisher Scientific) for 2 h. The sections were then stained with Hoechst 33342 (10 µg mL^−1^) for 20 min. The fluorescent images of the sections were captured on a Zeiss Axio Observer Z1 fluorescence microscope.

### Statistical Analysis

The results are expressed as means ± standard errors of the means (SEM) based on at least three independent experiments. The statistical differences were evaluated with one-way analysis of variance (ANOVA) followed by Tukey’s post hoc test. A *P* < 0.05 was considered as statistically significant.

## Results and Discussion

### Synthesis of the NBP@TiO_2_ Nanostructures

**Fig. 1:**
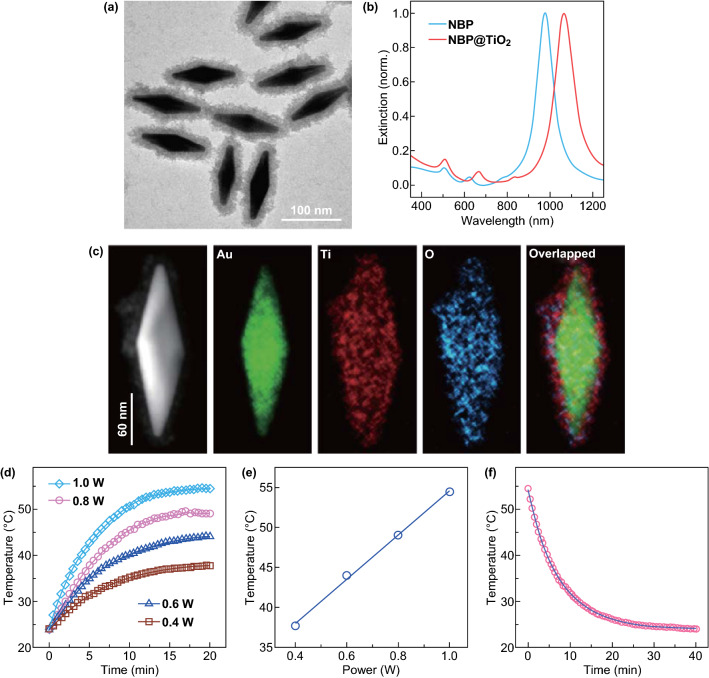
(Au NBP)@TiO_2_ nanostructures. **a** TEM image, **b** extinction spectra of the uncoated NBP and NBP@TiO_2_ nanostructures in aqueous solutions, **c** HAADF-STEM and elemental mapping images of a single NBP@TiO_2_. The rightmost image is the overlapped image of the Au, Ti, and O elemental maps, **d** temperature rise curves of the NBP@TiO_2_ nanostructures (2 mL, 120 μg mL^−1^) acquired under 1064-nm laser irradiation at different optical powers for 20 min, **e** variation of the reached plateau temperature with the laser power, **f** temperature decay curve. The data points (red circles) were measured during the cooling process after the NBP/TiO_2_ nanostructure solution (2 mL, 120 μg mL^−1^) was irradiated with a 1064-nm laser at 1.0 W for 20 min. The blue line was obtained from fitting. (Color figure online)

A seed-mediated growth method [30] was used to prepare the Au NBPs in aqueous solutions with cetyltributylammonium bromide (CTBAB) as the stabilizing agent. The raw NBPs were further purified following our reported protocol [22]. The purification procedure included Ag overgrowth, depletion force-induced self-separation, and final chemical etching of Ag [22]. The NBPs with number purities approaching 100% were obtained after purification. The NBPs were then coated with TiO_2_ by employing TiCl_3_ as the precursor [29]. The TEM image of the obtained NBP@TiO_2_ nanostructures is displayed in Fig. 1a. The prepared NBP@TiO_2_ nanostructures show great uniformity in shape and size. The NBP cores have a pentagonal base at the waist and two sharp apexes. They have an average length of 116.9 ± 1.1 nm and waist width of 32.9 ± 0.3 nm. The average thickness of the TiO_2_ coating layer is 16.6 ± 0.4 nm. The extinction profiles of the NBP and NBP@TiO_2_ samples are shown in Fig. 1b. The longitudinal plasmon wavelength of the NBP nanostructures is 970 nm. The TiO_2_ coating induces a redshift of the longitudinal plasmon resonance peak to 1064 nm, which enables the NBP@TiO_2_ nanostructures as a PTT agent in the NIR-II window. We also performed HAADF-STEM imaging and elemental mapping of the NBP@TiO_2_ sample. Elemental mapping (Fig. 1c) clearly shows the presence of Ti and O atoms on the surface of NBP.

### Photothermal Conversion Efficiency of the NBP@TiO_2_ Nanostructures

The photothermal conversion efficiency of the NBP@TiO_2_ nanostructures was first determined under 1064-nm laser irradiation. Figure 1d displays the temperature rise curves of the aqueous NBP@TiO_2_ solution under 1064-nm laser irradiation at various optical powers. The temperature of the solution quickly increases during 10 min and reaches a plateau at 20 min. The temperature reached at the plateau increases with the increment of the laser power. The end temperature reaches 37.7, 44.0, 49.0, and 54.4 °C at the optical power of 0.4, 0.6, 0.8, and 1.0 W, respectively (Fig. 1e). A reported theoretical model [21] was employed to calculate the photothermal conversion efficiency *η* according to Eq. 1:

1 $$ \eta = \frac{{B\left( {T_{\text{end}} - T_{0} } \right) + C\left( {T_{\text{end}} - T_{0} } \right)^{2} - I\xi }}{{I\left( {1 - \xi } \right)\left( {1 - 10^{ - E} } \right)}} $$

where *I* and *E* are the optical power and peak extinction value, respectively, *B* and *C* are two coefficients and determined from the cooling curve (Fig. 1f). The determined values of *B* and *C* are 1.7557 J K^−1^ min^−1^ and 0.00004 J K^−2^ min^−1^, respectively. *T*_0_ and *T*_end_ are the temperatures of the solution before and after laser irradiation, respectively. *ξ* is the fraction of the laser energy absorbed by the cuvette wall and the solution. This value was determined to be 0.1111 by measuring the temperature rise and decay curves of pure water. According to Eq. 1, the photothermal conversion efficiency of the NBP@TiO_2_ nanostructures was determined to be (93.3 ± 5.2)% under 1064-nm laser irradiation.

### CA4P Loading

Endothelial cells line at the interior surface of blood vessels. They supply oxygen and nutrients for tissues and organs [31]. Because tumors need to rapidly develop new vascular networks to support the high proliferation of cancer cells, tumor blood vessels are therefore clearly a target for cancer therapy [26]. Antiangiogenesis therapy can prevent the formation of new blood vessels or disrupt the existent vasculatures in tumors, hence blocking the transport of nutrients and oxygen from blood vessels to tumor tissues, and finally cause tumor necrosis in a large area [32].

**Fig. 2:**
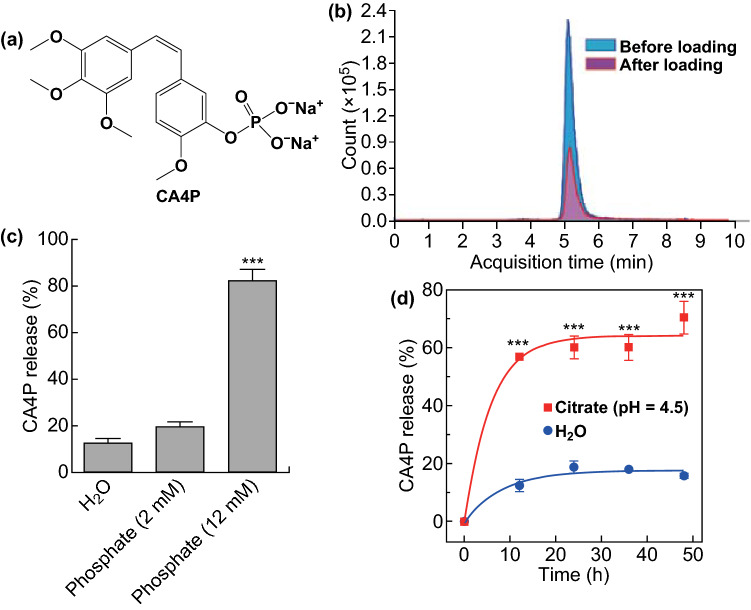
NBP@TiO_2_ nanostructures for CA4P loading. **a** Molecular structure of CA4P, **b** LC–MS chromatograms of CA4P for the initial CA4P solution and the supernatant solution after drug loading, **c** desorption of CA4P in phosphate solutions and H_2_O after 12-h incubation, **d** pH- and time-dependent CA4P release profiles for the CA4P-loaded NBP@TiO_2_ nanostructures. The CA4P-loaded NBP/TiO_2_ nanostructures (120 μg Au) were dispersed in phosphate solutions (2 and 12 mM PO_4_^3−^, 1 mL), citrate buffer (20 mM, pH 4.5, 1 mL), or H_2_O (1 mL) and incubated at 37 °C. The CA4P release percentage was calculated by measuring the drug concentration in the supernatant. The shown data represent the mean ± SEM. ***P < 0.001

CA4P (Fig. 2a) can selectively bind to tubulin, resulting in microtubule depolymerization, triggering morphological changes in endothelial cells, and therefore causing the shutdown of tumor blood vessels [33, 34]. However, CA4P has cardiovascular toxicity, which limits its further clinical applications [35].

We designed a multifunctional system for combined chemotherapy and PTT. In this system, CA4P was loaded onto the NBP@TiO_2_ nanostructures, because phosphate has a high binding affinity to titania [36]. Such a system is capable of delivering both CA4P and hyperthermia to tumors.

For the drug loading study, the NBP@TiO_2_ nanostructures were mixed with a CA4P solution, and the mixture was stirred overnight. After centrifugation, the concentration of the supernatant was determined by LC–MS. As shown in Fig. S1, the calibration curve of CA4P shows a good linearity at the concentration of 0–17.5 μM, indicating that the measurement method is reliable. Compared with the initial CA4P solution, the drug concentration in the supernatant solution after drug loading decreases dramatically, suggesting that most of the drug is adsorbed by the NBP@TiO_2_ nanostructures (Fig. 2b). The drug encapsulation efficiency for the NBP@TiO_2_ nanostructures was calculated to be (64.8 ± 6.8)%. The drug loading capacity of CA4P on the NBP/TiO_2_ nanostructures was calculated to be 8.3 wt% according to the encapsulation efficiency of 64.8% above. The FTIR spectrum of the drug-loaded NBP@TiO_2_ is presented in Fig. S2a. Compared with the NBP@TiO_2_ sample, the CA4P-loaded NBP@TiO_2_ sample shows IR peaks at 3386, 2937, 2836, 1579, 1512, 1269, 1236, 1122, 1055, 983, 856, 792, and 744 cm^−1^, which are attributed to CA4P. TGA result (Fig. S2b) also confirmed the presence of additional drug on the NBP@TiO_2_ nanostructures. If the concentration of CA4P in the solution is reduced to 35 μM, the encapsulation efficiency is (98.6 ± 0.4)%.

The pharmacological effects of CA4P in different models are highly dependent on its dosage. A high dosage is required to induce cell death, while a low dosage is able to interrupt intracellular tubulin distribution and inhibit angiogenesis. Considering the drug encapsulation efficiency approaches to 100% when using the CA4P solution at a low concentration (< 35 μM), the ratio of CA4P to NBP@TiO_2_ nanostructures can be controlled.

### Drug Release

Controlled-drug release from drug-loaded nanoparticles is a key factor for exerting their therapeutic effects. In order to evaluate the binding affinity of CA4P to TiO_2_, we used phosphate buffer solutions (2 and 12 mM PO_4_^3−^) to competitively displace CA4P molecules from the surface of TiO_2_. (82.1 ± 5.0)% of CA4P was stripped from the TiO_2_ surface in the presence of 12 mM PO_4_^3−^, as shown in Fig. 2c. However, only (12.5 ± 2.0)% and (19.6 ± 2.0)% of CA4P desorbed from the NBP@TiO_2_ nanostructures in H_2_O and the phosphate buffer solution containing PO_4_^3−^ at 2 mM, respectively (Fig. 2c). This result demonstrates that the high loading capacity of the NBP@TiO_2_ nanostructures for CA4P is caused by a high binding affinity between the phosphate group of CA4P and TiO_2_. Because the serum phosphate concentration is less than 2 mM in humans, our result suggests that a limited amount of CA4P will be released from the drug-loaded nanostructures when they are injected. As cell-internalized nanoparticles are mainly distributed in acidic endosomes/lysosomes [37], we also studied the CA4P drug release in an endosome/lysosome mimicking buffer (20 mM citrate, pH 4.5). Release of CA4P at (70.3 ± 5.6)% from the CA4P-loaded NBP@TiO_2_ nanostructures was observed after incubation for 48 h (Fig. 2d). On the other hand, laser irradiation at 1064 nm (1.6 W cm^−2^, 3 min) does not considerably affect the CA4P release from the drug-loaded nanoparticles in water (data not shown).

### PTT Study in A549 Cells

**Fig. 3:**
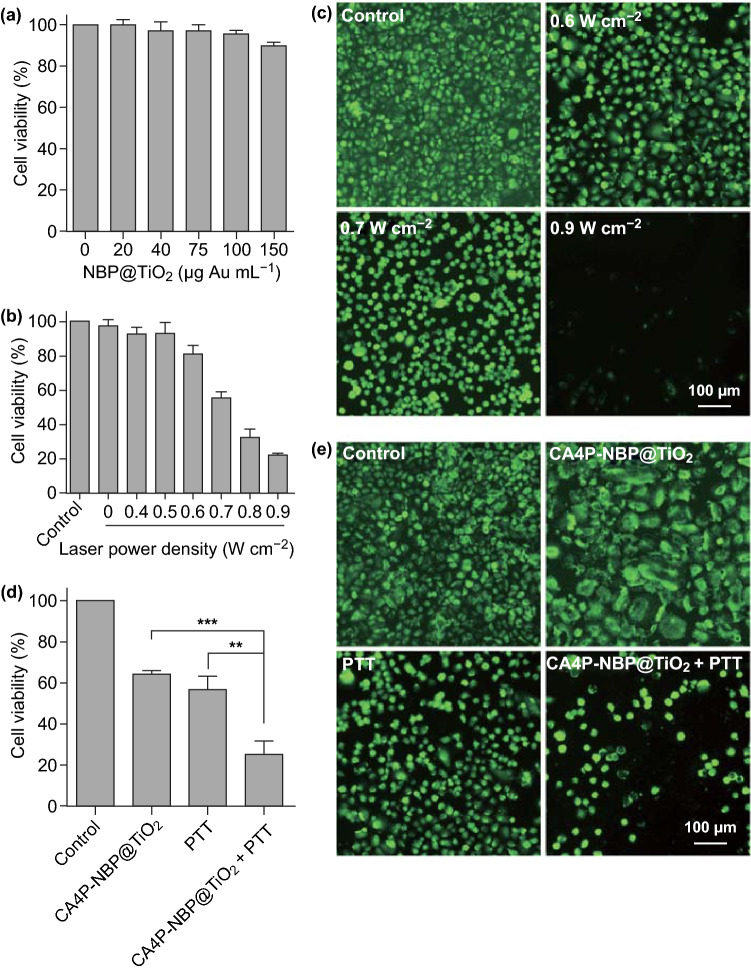
PTT in A549 cells. **a** Effect of the NBP@TiO_2_ nanostructures at different concentrations on the viability of A549 cells, **b** cell viabilities of A549 cells upon PTT at different optical power densities, **c** calcein AM staining of the cells treated by PTT at different optical power densities as in **b**. A549 cells were incubated with the NBP@TiO_2_ nanostructures (100 μg-Au mL^‒1^) for 24 h, followed by 1064-nm laser irradiation at 0.4‒0.9 W cm^‒2^ for 5 min. The live cells were stained with green fluorescence by calcein AM, **d** synergistic enhancement of the cytotoxicity of the CA4P-loaded NBP@TiO_2_ nanostructures by combined chemotherapy and PTT, **e** calcein AM staining of the A549 cells after the different therapy treatments as in **d**. A549 cells were treated with the CA4P-loaded NBP@TiO_2_ nanostructures (15 nM CA4P, 100 μg-Au mL^‒1^) or together with 1064-nm laser irradiation at 0.7 W cm^‒2^ for 5 min. The data shown represent the mean ± SEM. **P < 0.01, ***P < 0.001

The effect of the NBP@TiO_2_ nanostructures on cell viability was examined in A549 cells using the MTT assay. As shown in Fig. 3a, the cell viability after the treatment with the NBP@TiO_2_ nanostructures remains above 95% even at a gold concentration up to 150 μg mL^−1^. This result indicates that the NBP@TiO_2_ nanostructures possess good biocompatibility.

Sole 1064-nm laser irradiation in the absence of the NBP@TiO_2_ nanostructures does not impair cells, as the cell viability remains the same as the control group. Due to the efficient light-to-heat conversion by the NBP@TiO_2_ nanostructures, 1064-nm laser irradiation on the cells in the presence of the NBP@TiO_2_ nanostructures (100 μg-Au mL^−1^) results in remarkable cytotoxicity, which is dependent on the laser power density. The survival rates of the cells decline to (81.1 ± 4.9)%, (55.8 ± 3.3)%, (32.1 ± 5.0)%, and (22.1 ± 0.4)% at laser power densities of 0.6, 0.7, 0.8, and 0.9 W cm^−2^, respectively (Fig. 3b). Calcein AM staining (Fig. 3c) confirmed this result. The live cells are stained with green fluorescence. Cell rounding is observable for most of the cells after the laser irradiation at a power density of 0.7 W cm^−2^. Nearly all of the cells are dead after the laser irradiation at 0.9 W cm^−2^.

For the combined chemo-photothermal therapy, the laser irradiation at a power density of 0.7 W cm^−2^ was employed. PTT in the presence of 100 μg-Au mL^−1^ of the NBP@TiO_2_ nanostructures reduces the cell viability to (56.7 ± 3.7)% (Fig. 3d). The viability of the cells treated with the CA4P-loaded NBP@TiO_2_ nanostructures (15 nM CA4P, 100 μg-Au mL^−1^) for 48 h is (64.3 ± 0.9)%. However, the combined chemo-photothermal therapy using the CA4P-loaded NBP@TiO_2_ nanostructures is much more effective than each therapy alone, and the cell viability remarkably declines to (25.3 ± 3.7)% (Fig. 3d). The different cell viabilities caused by the different therapies were also verified by calcein AM staining (Fig. 3e). The treatment with the CA4P-loaded NBP@TiO_2_ nanostructures (15 nM CA4P, 100 μg-Au mL^−1^) induces clear cell swelling. The cells treated with the combined chemo-photothermal therapy show a clear round-up morphology. This result indicates that PTT and chemotherapy exert a synergistic anticancer effect.

### PTT Effect on HUVECs

Hyperthermia therapy has been reported to inhibit angiogenesis [38, 39]. Therefore, the PTT effect of the NBP@TiO_2_ nanostructures on the endothelial cell activity was assessed. As shown in Fig. S3a, the NBP@TiO_2_ nanostructures (48 μg-Au mL^−1^) do not affect the viability of HUVECs. In addition, the NBP@TiO_2_ nanostructures are readily internalized by HUVECs, as confirmed by the accumulation of clear brown NBP@TiO_2_ granules at the cytoplasm of the HUVECs after the cells were treated with the NBP@TiO_2_ nanostructures (12 μg-Au mL^−1^) for 48 h (Fig. S3b).

In order to reduce the influence of the NBP@TiO_2_ nanostructures on fluorescence imaging, a low dosage (12 μg-Au mL^−1^) was used in this study. The PTT effect of the NBP@TiO_2_ nanostructures under 1064-nm laser irradiation for 3 min was examined in HUVECs. As shown in Fig. S4a, the cell viability decreases as the laser irradiation power density is increased in the presence of the NBP@TiO_2_ nanostructures (12 μg-Au mL^−1^). Compared with the control group, the cell viabilities are reduced to (86.9 ± 2.6)%, (74.1 ± 4.7)%, (44.2 ± 2.6)%, and (25.7 ± 0.8)% at optical power densities of 1.5, 1.8, 2.0, and 2.3 W cm^−2^, respectively. Calcein AM staining (Fig. S4b) also confirmed this result. Cell rounding occurs for most of the cells after the laser irradiation at 1.8 W cm^−2^ in the presence of the NBP@TiO_2_ nanostructures. Most of the cells are dead after the laser irradiation at 2.3 W cm^−2^.

**Fig. 4:**
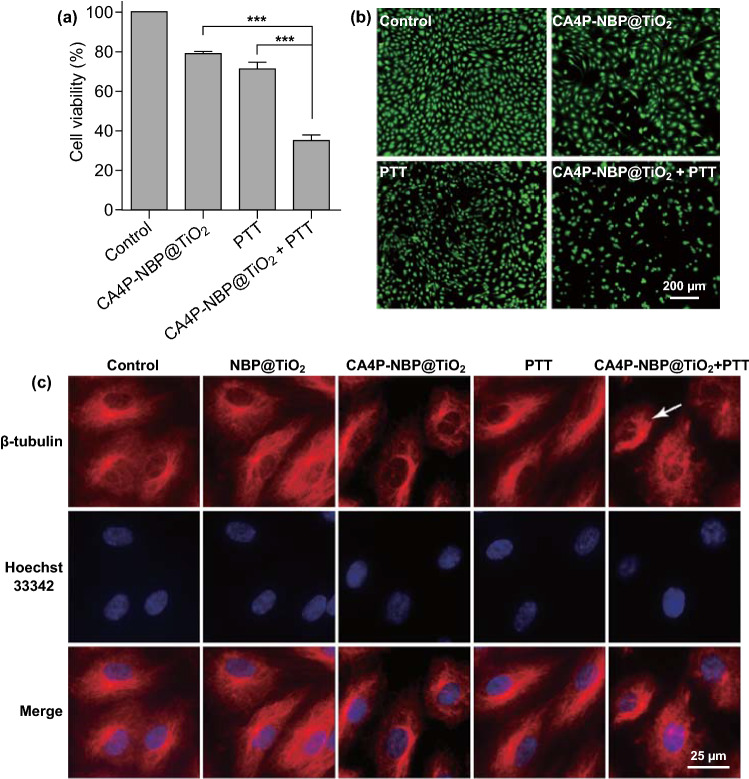
PTT-enhanced cytotoxic effect of the CA4P-loaded NBP@TiO_2_ nanostructures in HUVECs. **a** MTT assay, **b** calcein AM staining. The HUVECs were treated with the CA4P-loaded NBP@TiO_2_ nanostructures (7 nM CA4P, 12 μg-Au mL^−1^) for 24 h and then exposed to 1064-nm laser irradiation at 1.8 W cm^−2^ for 3 min. The assay and staining were performed after further incubation for 24 h. The shown data represent the mean ± SEM. ***P < 0.001, **c** synergistic disruption of tubulin in the HUVECs by the combined therapy. Immunofluorescent staining with β-tubulin (red) was performed on the HUVECs treated with the CA4P-loaded NBP@TiO_2_ nanostructures (3 nM CA4P, 12 μg-Au mL^−1^) in conjunction with 1064-nm laser irradiation (1.3 W cm^−2^, 3 min). The nuclei were stained with Hoechst 33342 (blue). In the control group, the tubulin shows a filamentous morphology. CA4P treatment induces a disordered change of tubulin and rounding of the cell morphology. The combined treatment further disrupts tubulin distribution and cell morphology (indicated by the white arrow). (Color figure online)

The efficacy of the CA4P-loaded NBP@TiO_2_ nanostructures in combination with PTT was also investigated in HUVECs. A low CA4P dose (CA4P-loaded NBP@TiO_2_ nanostructures, 7 nM CA4P, 12 μg-Au mL^−1^), and a low laser irradiation dose (1.8 W cm^−2^, 3 min) were employed in this experiment. The cell viabilities after the two separate treatments are (79.1 ± 0.7)% and (71.2 ± 3.4)%, respectively. However, a combination of the CA4P-loaded NBP@TiO_2_ nanostructures with PTT reduces the cell viability to (35.2 ± 2.7)% in a synergistic manner (Fig. 4a). In the calcein AM staining analysis (Fig. 4b), almost all of the cells become rounded and green fluorescence is not detectable for most cells after the combined chemo-photothermal therapy. These results indicate that the simultaneous treatment with the CA4P-loaded NBP@TiO_2_ nanostructures and PTT induces a more severe damage to HUVECs than either therapy alone.

### PTT-Enhanced Tubulin Disruption

Microtubules are highly dynamic components of the cytoskeleton that play vital roles in cell proliferation, trafficking, signaling and migration [40]. They are formed by the polymerization of a dimer of α- and β-tubulin. CA4P is a prodrug. It can be rapidly converted to the active form, which has a high binding affinity to tubulin. The intracellular target for CA4P is therefore tubulin. In this regard, the effect of the combined chemo-photothermal therapy with the CA4P-loaded NBP@TiO_2_ nanostructures on microtubules was investigated. Neither CA4P at a low dosage (3 nM) nor a low-power laser irradiation (1.3 W cm^−2^, 3 min) mediated by the NBP@TiO_2_ nanostructures (12 μg-Au mL^−1^) affects the intracellular tubulin distribution as revealed by immunofluorescent imaging in Fig. 4c. However, the combined treatment with the CA4P-loaded NBP@TiO_2_ nanostructures together with PTT causes strong disruption of the endothelial tubulin distribution (Fig. 4c).

### PTT-Enhanced Antiangiogenesis Effect of CA4P

A critical step in tumor development and metastasis is angiogenesis, which supplies nutrients and oxygen and removes waste products for cells. Endothelial cell proliferation, migration, and capillary tube formation are important events during angiogenesis. When HUVECs are plated on a basement membrane matrix (Matrigel) in the presence of growth factors in short-term culture, they align into the networks of tubules. CA4P can disrupt the tubular structure and hence inhibit the branching outgrowth of endothelial cells [41]. We therefore further evaluated whether the low-power laser irradiation can inhibit HUVEC angiogenesis.

**Fig. 5:**
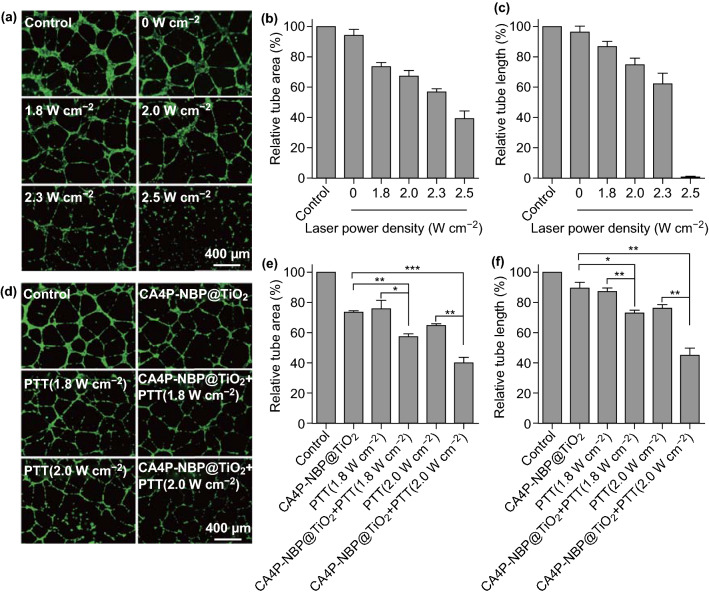
Inhibition of the endothelial tube formation by the combined chemo-photothermal therapy using the CA4P-loaded NBP@TiO_2_ nanostructures. **a** Effect of PTT at different laser power densities (1.8–2.5 W cm^−2^) on the HUVEC tube formation, **b** relative tube areas estimated from **a**, **c** relative tube lengths estimated from **a**, **d** PTT-enhanced antiangiogenesis of the CA4P-loaded NBP@TiO_2_ nanostructures, **e** relative tube areas estimated from **d**, **f** relative tube lengths estimated from **d**. The HUVECs were seeded in a 48-well plate that was pre-coated with Matrigel and then treated with the NBP@TiO_2_ nanostructures (12 μg-Au mL^−1^) or the CA4P-loaded NBP@TiO_2_ nanostructures (7 nM CA4P, 12 μg-Au mL^−1^), followed by 1064-nm laser irradiation for 3 min. The tubular structures were visualized with calcein AM staining, and the relative tube areas and lengths were calculated. The as-shown results are the mean ± SEM. *P < 0.05, **P < 0.01, ***P < 0.001

As shown in Fig. 5a, in the control group, the HUVECs differentiate into a well-defined tube-like network on the Matrigel after 12-h incubation. The quantitative analysis for the tube area and the tube length was conducted by an Image-Pro Plus 6.0 software, as shown in Fig. 5b, c. The treatment with the NBP@TiO_2_ nanostructures (12 μg-Au mL^−1^) has no effect on the tube formation. As shown in Fig. 5a, after the PTT treatment (1.8–2.3 W cm^−2^), the HUVECs do not completely differentiate and instead only form incomplete, short, and thin tubes. Compared with the control group, the tube areas decrease to (73.6 ± 2.6)%, (67.1 ± 3.9)%, and (57.0 ± 1.9)% at optical power densities of 1.8, 2.0, and 2.3 W cm^−2^, respectively. The tube lengths correspondingly decrease to (86.6 ± 3.6)%, (74.6 ± 4.4)%, and (62.1 ± 7.1)% at optical power densities of 1.8, 2.0, and 2.3 W cm^−2^. Irradiation at 2.5 W cm^−2^ completely disrupts the formation of the capillary-like structure.

The CA4P-loaded NBP@TiO_2_ nanostructures at a low dose (7 nM CA4P, 12 μg-Au mL^−1^) slightly inhibit the HUVEC tube formation, as the relative tube area and tube length are (73.7 ± 1.0)% and (89.6 ± 3.8)%, as compared with the control group (Fig. 5d–f). Interestingly, the combined treatment using the CA4P-loaded NBP@TiO_2_ nanostructures under the laser irradiation at a low power density (1.8 or 2.0 W cm^−2^) synergistically inhibits the tube formation. The tube area and tube length are (57.5 ± 2.0)% and (73.2 ± 1.8)% in the combined therapy using the laser irradiation at 1.8 W cm^−2^. These two values are decreased to (40.1 ± 3.6)% and (45.2 ± 4.6)% under the laser irradiation at 2.0 W cm^−2^ (Fig. 5d–f).

### In Vivo PTT

In the in vivo study, the NBP@TiO_2_ nanostructures were coated with PEG to improve their colloidal stability. PEG molecules functionalized with dopamine at one end bind to the surface of TiO_2_ through the bidentate coordination of the dopamine OH groups to the under-coordinated Ti surface atoms [42, 43]. The obtained NBP@TiO_2_ nanostructures with PEG coating show a high colloidal stability even in the saline solution (0.9% NaCl) (Fig. S5a). Interestingly, the binding of the dopamine-functionalized PEG does not competitively desorb CA4P from the CA4P-loaded NBP@TiO_2_ nanostructures. As shown in Fig. S5b, after the coating of the dopamine-functionalized PEG, the CA4P release from the drug-loaded nanostructures is determined to be (6.9 ± 0.3)%, which is even less than that in water. The CA4P-loaded NBP@TiO_2_ nanostructures are also colloidally stable in 0.9% NaCl or PBS, even after 5-day incubation (Fig. S6).

Biocompatibility study was firstly performed. Mice were intravenously injected with NBP@TiO_2_ nanostructures (25 mg-Au kg^−1^) and then killed after 20 days, with their blood and main organs harvested for biocompatibility study. Blood analysis suggested that NBP@TiO_2_ nanostructures do not have effect on complete blood count (Table S1). Hematoxylin and eosin (H&E) staining also evidenced that NBP@TiO_2_ nanostructures show no noticeable organ damage or inflammatory (Fig. S7). It was also found that NBP@TiO_2_ nanostructures are mainly accumulated in the liver at 1-day post-intravenous injection (Fig. S8), because large particles can be rapidly cleared by the reticuloendothelial system [44].We carried out the in vivo study using an intratumoral administration.

**Fig. 6:**
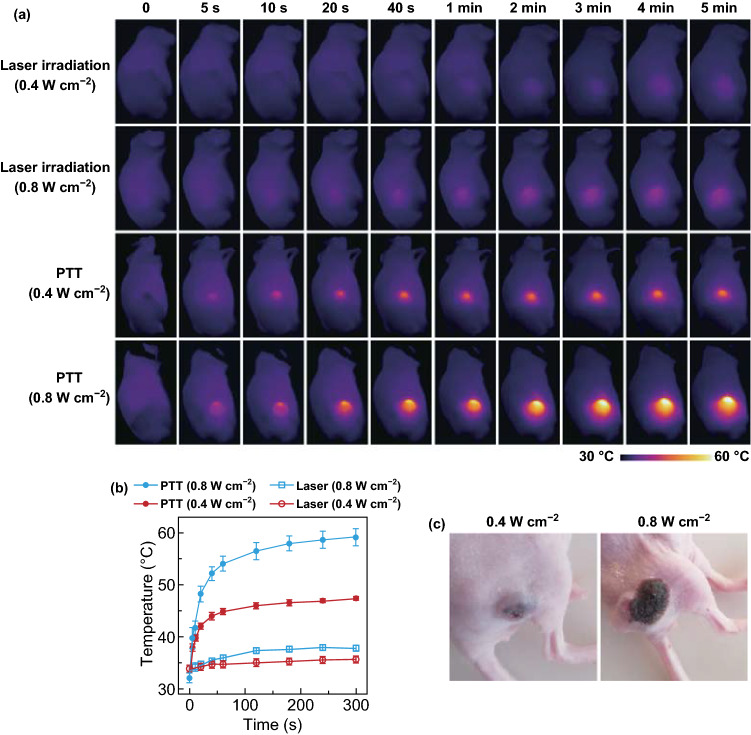
In vivo PTT. **a** Infrared thermal images of the A549-bearing mice, **b** tumor temperature change curves extracted from **a**. The A549-bearing mice intratumorally injected with the NBP@TiO_2_ nanostructures were subjected under 1064-nm laser irradiation at 0.4 or 0.8 W cm^−2^ for 5 min. The mice without the administration of the NBP@TiO_2_ nanostructures were subjected to the laser irradiation as control. The as-shown results are the mean ± SEM, **c** photographs of the tumor captured at 48 h after PPT at 0.4 or 0.8 W cm^−2^

At 24 h after injection, the tumor region was irradiated with the 1064-nm laser, and an infrared thermal camera was used to monitor the temperature change (Fig. 6a). The above in vitro PTT studies were performed at room temperature (~ 25 °C). A much lower irradiation power was needed to reach the equal hyperthermia level for the in vivo study, as the temperature of the tumor is ~ 34 °C. In the control mice without the injection of the NBP@TiO_2_ nanostructures, 1064-nm laser irradiation at 0.4 or 0.8 W cm^−2^ for 5 min induces negligible temperature increases in the tumor area. Compared with the initial temperature, the temperature increments (Δ*T*) are only 1.6 ± 0.4 and 4.0 ± 0.7 °C, respectively (Fig. 6a, b). Such temperatures are not high enough to destroy the tumor [45, 46]. For the mice injected with the NBP@TiO_2_ nanostructures (25 mg-Au kg^−1^), the temperature of the tumor steadily increases to 47.4 ± 0.3 °C (Δ*T* = 13.6 ± 0.7 °C) within 5 min during the exposure to 1064-nm laser irradiation at the power density of 0.4 W cm^−2^. This power density is notably much lower than the maximal permissible exposure value (1.0 W cm^−2^) under 1064-nm laser irradiation [5]. Moreover, the surrounding region near the tumor shows a negligible temperature increase. Irradiation at the power density of 0.8 W cm^−2^ at the tumor injected with the NBP@TiO_2_ nanostructures induces a dramatic temperature increase immediately, even as early as at 20 s after the start of laser irradiation. The temperature at the center of the tumor reaches 59.2 ± 1.7 °C (Δ*T* = 27.1 ± 1.6 °C) after irradiation for 5 min. However, the generated heat diffuses to the nearby normal tissues. Irradiation at this power density causes burning, blistering, and pain to the mouse during the treatment. In fact, how to provide useful therapeutic outcomes without photo-induced skin damage has remained challenging for PTT. The pictures of the tumor skin were captured at 2 days after PTT (Fig. 6c). No skin damage was observed after irradiation at 0.4 W cm^−2^. However, at 2 days after PTT at 0.8 W cm^−2^, a broad black scar covering the tumor and the nearby normal tissues appears due to the severe heat damage (Fig. 6c).

### NBP@TiO_2_ Nanostructures as an X-ray CT Contrast Enhancer

**Fig. 7:**
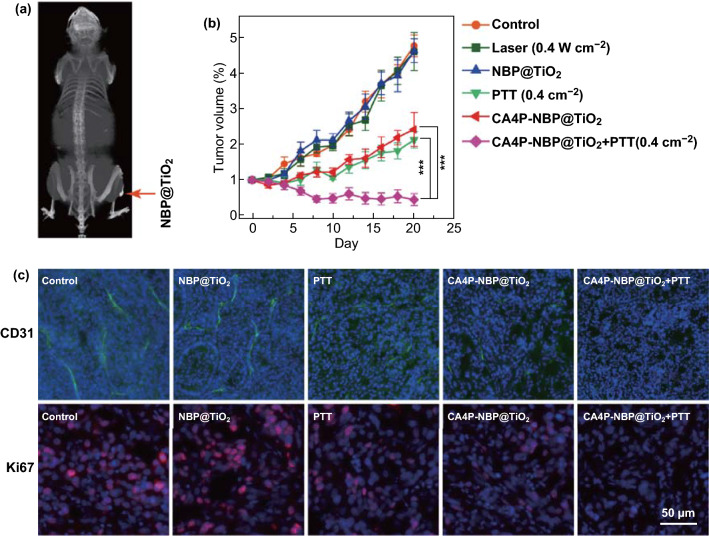
In vivo antitumor effect. **a** Typical three-dimensional reconstructed CT image of an A549 tumor-bearing mouse after intratumoral injection with the NBP@TiO_2_ nanostructures. The arrow head points to the tumor site, **b** tumor growth in the different groups at different time points. For the laser irradiation treatment groups, the mice were intratumorally injected with the NBP@TiO_2_ (25 mg-Au kg^−1^) or the CA4P-loaded NBP@TiO_2_ nanostructures (25 mg-Au kg^−1^, 2 mg CA4P kg^−1^). At 24 h after the injection, the mice were subjected to 1064-nm laser irradiation (0.4 W cm^−2^, 5 min). The as-shown results are the mean ± SEM. ***P < 0.001, *n* = 6, **c** immunofluorescence images. The immunofluorescence staining of the tumor sections was performed to image the tumor micro-vessel density with anti-CD31 (green) and to evaluate cell proliferation with anti-Ki67 (red) at the tumor center. The nuclei were stained with Hoechst 33342 (blue). (Color figure online)

High-atomic-number elements such as Au have shown an excellent X-ray attenuation ability. Au nanostructures have been considered as the contrast-enhancing agents for CT imaging, which is widely used for medical diagnosis due to its deep tissue penetration and high-resolution imaging quality [47]. We also investigated the NBP@TiO_2_ nanostructures as a contrast-enhancing agent for CT imaging. After the intratumoral injection of the PEG-coated NBP@TiO_2_ nanostructures (25 mg-Au kg^−1^), the reconstructed three-dimensional CT image of the nude mouse clearly displays contrast in the tumor region (Fig. 7a), revealing that the NBP@TiO_2_ nanostructures can be simultaneously used as a CT contrast-enhancing agent for tumor imaging.

### In Vivo Combined Chemo-Photothermal Therapy

Inspired by the excellent in vitro performance of the CA4P-loaded NBP@TiO_2_ nanostructures, the in vivo antiangiogenesis and antitumor activities of the CA4P-loaded NBP@TiO_2_ nanostructures were assessed in A549-tumor-bearing mice. Six groups of mice were treated as follows: Group I, control; Group II, 1064-nm laser irradiation only; Group III, NBP@TiO_2_ nanostructures; Group IV, PTT, NBP@TiO_2_ nanostructures + 1064-nm laser irradiation; Group V, chemotherapy, CA4P-loaded NBP@TiO_2_ nanostructures; Group VI, PTT + chemotherapy, CA4P-loaded NBP@TiO_2_ nanostructures + 1064-nm laser irradiation. The NBP@TiO_2_ and CA4P-loaded NBP@TiO_2_ nanostructures were administered into the mice with an intratumoral injection. The dosage was 25 mg-Au kg^−1^ for the NBP/TiO_2_ or the CA4P-loaded NBP@TiO_2_ nanostructures, and 2 mg-CA4P kg^−1^ for the drug-loaded nanostructures.

The in vivo antitumor efficacy was finally investigated by monitoring the volume of the tumor in 20 days after the treatment. The treatment does not affect the body weight of mice (Fig. S9). As shown in Fig. 7b, the laser irradiation only and the NBP@TiO_2_ nanostructures exhibit no effect on the tumor growth in comparison with the control group. The tumor volumes of these three groups increase to ~ 4.7-fold the original ones.

The tumor growth is delayed in the mice of the PTT and CA4P groups. The mice treated with PTT (Group IV) and chemotherapy (Group V) exhibit 55.4% and 49.1% inhibition on the tumor growth as compared with the control (Group I), respectively. Moreover, the laser irradiation density (0.4 W cm^−2^) used in our study is quite low, but the tumor growth is significantly inhibited due to the high photothermal conversion efficiency of the NBP@TiO_2_ nanostructures.

The combined treatment of the CA4P-loaded NBP@TiO_2_ nanostructures and 1064-nm laser irradiation (Group VI) is the most effective in destructing the tumor. The tumor shrinks remarkably along with time, and the tumor volume declines continuously to 0.4-fold its original one at 20 day after the treatment. In contrast, the tumor volumes of the groups treated with PTT and CA4P increase to ~ 2.1 and 2.4-fold the original ones, respectively.

We next examined whether the treatments cause histological changes in the tumor tissues. CD31 is a characteristic surface marker of endothelial cell lineage. We therefore used CD31 immunostaining to observe the vasculatures in the tumors. Larger vessels are distributed at the edge of the tumors (Fig. S10), as compared with that in the tumor center (Fig. 7c). The tumors in both control and NBP@TiO_2_ nanostructure-treated groups maintain the apparent normal vasculature structure in the tumor center (Fig. 7c). PTT at 0.4 W cm^−2^ and CA4P (2 mg kg^−1^) significantly reduce the micro-vessel density. The combined therapy fully disrupts the vasculature in the entire tumor (Figs. 7c and S10). The tumor cellular proliferative activity was measured by Ki67 immunostaining. The control and NBP@TiO_2_ nanostructure-treated groups show a high proliferative activity according to the strong staining of Ki67. Similar to the CD31-staining result, the combined chemo-photothermal therapy induces the maximal inhibition of Ki67 expression in the tumor, indicating a significant reduced proliferation of the tumor cells after the combined therapy. These results further confirm that the combined therapy using the CA4P-loaded NBP@TiO_2_ nanostructures under 1064-laser irradiation has the highest inhibition effect on the tumor growth in vivo.

## Conclusions

Exploration of photothermal agents with high photothermal conversion efficiencies is of great importance for maximizing the in vivo PTT efficacy and compensating for the adverse effects caused by the use of laser irradiation energies that are as low as possible. Low efficiencies in light-to-heat conversion are the primary barrier for most photothermal agents that are responsive in the NIR-II window. With photothermal agents of low photothermal conversion efficiencies, an ideal therapeutic outcome can only be obtained by use of skin-harmfully high laser irradiation. Photothermal agents with high photothermal conversion efficiencies in the NIR-II region are in strong demand. In this work, we synthesized the NBP@TiO_2_ nanostructures with a photothermal conversion efficiency of (93.3 ± 5.2)% at the wavelength of 1064 nm. This value is the highest among the reported photothermal agents responsive in the NIR-II window. This can probably be attributed to the fact that the longitudinal plasmon wavelength of the NBP@TiO_2_ nanostructures is equal to the wavelength of the irradiating laser [21]. As a result, the NBP@TiO_2_ nanostructures are expected to be an ideal PTT agent in the NIR-II window.

Angiogenesis has become a target for cancer therapy. CA4P-based antiangiogenesis therapy has been in clinical development for cancer therapy. However, the antitumor efficacy is unsatisfactory if only the antiangiogenesis therapy is adopted, and CA4P has clear cardiovascular toxicity [35]. To overcome the drawbacks of chemotherapy, the design and construction of multifunctional targeting drug delivery systems is one of the effective and prevailing approaches. In this work, we designed a novel multifunctional CA4P delivery system for combined chemotherapy and PTT. Due to a high binding affinity between the phosphate group of CA4P and TiO_2_, the NBP@TiO_2_ nanostructures have a high loading capacity of 8.3 wt% for CA4P. This value is very high among Au nanostructure-based drug delivery systems.

Photothermal agents loaded with anticancer drugs can deliver both heat and drugs simultaneously to tumors. Furthermore, hyperthermia induced by PTT can also synergistically enhance the anticancer effects of chemotherapeutic agents [48–50] and reduce their side effects [51, 52]. The in vitro results reveal that PTT synergistically enhances the anticancer and antiangiogenesis effects of CA4P. We believe that this combined therapy will improve the therapeutic effect of CA4P while reducing its side effects because a lower dosage is needed in the combined therapy.

The high temperature induced by PTT is key to the antitumor efficacy. In order to achieve a good hyperthermia effect, an elevated tumor-killing hyperthermia temperature (> 50 °C) is commonly used. But the high temperature inevitably induces heating damage to the surrounding normal tissues and even exceeds the patient’s tolerance. The high temperature-induced cell necrosis can also cause inflammation. On the other hand, because of the limited in vivo light penetration, it might not be realistic to deliver sufficient heating to deeply seated tumors even when using low-temperature (~ 45 °C) hyperthermia therapy, as some cancer cells can survive after PTT and spread out to other organs later. Achieving effective tumor killing under relatively low temperatures is therefore crucial for the successful application of PTT. Tremendous efforts are being made to destruct tumors through low-temperature PTT through the incorporation of chemotherapy.

The in vivo studies reveal that the CA4P-loaded NBP@TiO_2_ nanostructures under mild 1064-nm laser irradiation at a power density of 0.4 W cm^−2^ are the most effective in destructing the tumor. The combined therapy fully disrupts the vasculature in the entire tumor and maximally inhibits the proliferation of the tumor cells. In addition, the power density used is notably much lower than the maximal permissible exposure value (1.0 W cm^−2^) under 1064-nm laser irradiation [5]. We believe that without compromising the therapeutic benefit, the dosage of CA4P can be reduced through the incorporation of NIR irradiation-assisted therapy. This combined therapy will be able to reduce the systemic side effects of CA4P.

Taken together, our results clearly show that the NBP@TiO_2_ nanostructures, which are responsive in the NIR-II window, are able to delivery both hyperthermia and CA4P to tumors. The combined chemo-photothermal therapy mediated by the CA4P-loaded NBP@TiO_2_ nanostructures is of great potential for cancer therapy in the future.

## Electronic supplementary material

Below is the link to the electronic supplementary material.

Supplementary material 1 (PDF 867 kb)
