# Supplementary material for: Antiangiogenesis-Combined Photothermal Therapy in the Second Near-Infrared Window at Laser Powers Below the Skin Tolerance Threshold
Source: Nanomicro Lett. 2019 Oct 31;11:93. doi: 10.1007/s40820-019-0327-4 (PMC7770887; doi:10.1007/s40820-019-0327-4)
Supplement: Supplementary file 1 — Supplementary material 1 (PDF 867 kb) [file 40820_2019_327_MOESM1_ESM.pdf]

Supporting Information for

Antiangiogenesis-Combined Photothermal Therapy in the Second Near-Infrared Window at Laser Powers Below the Skin Tolerance Threshold

Jian-Li Chen[1], Han Zhang[2], Xue-Qin Huang[1], Hong-Ye Wan[1], Jie Li[1], Xing-Xing Fan[1], Kathy Qian Luo[3], Jinhua Wang[4], Xiao-Ming Zhu[1,]*, Jianfang Wang[2,]*

[1]State Key Laboratory of Quality Research in Chinese Medicine, Macau Institute for Applied Research in Medicine and Health, Macau University of Science and Technology, Taipa, Macau SAR, People's Republic of China

[2]Department of Physics, The Chinese University of Hong Kong, Shatin, Hong Kong SAR, People's Republic of China

[3]Faculty of Health Sciences, University of Macau, Taipa, Macau SAR, People's Republic of China

[4]Beijing Key Laboratory of Drug Targets Research and New Drug Screening, Institute of Materia Medica, Chinese Academy of Medical Sciences and Peking Union Medical College, Beijing 100050, People's Republic of China

*Corresponding authors. E-mail: xmzhu@must.edu.mo (X.-M. Zhu); jfwang@phy.cuhk.edu.hk (J. F. Wang)

S1 Experimental

S1.1 Analysis of Au Content in the Organs

The mice were injected in the tail vein with PEG-coated NBP@TiO_2 nanostructures (25 mg-Au kg^{-1}). The mice were then sacrificed after 24 h, and the organs (heart, liver, spleen, lung, kidney and tumors) were collected and weighed. The organs were cut into small pieces and dried at elevated temperature. The obtained dry mass was dissolved in 3 mL of aqua regia in order to convert metallic Au to Au(III) chloride irons. The obtained solution was further diluted with H_2O, and next analyzed on ICP-AES.

S1.2 Biocompatibility Study

The mice were injected in the tail vein with PEG-coated NBP@TiO_2 nanostructures (25 mg-Au kg^{-1}), and sacrificed after 20 days. The blood was collected, and the complete blood count was measured by a Nihon Kohden MEK-6318K hematology analyzer. The major organs from those mice, including heart, liver, spleen, lung and kidney, were collected for histology analysis. Before the histology analysis, these organs were fixed in 4% PFA and processed into paraffin, followed by sectioning and staining with H&E.

S2 Supplementary Figures and Tables

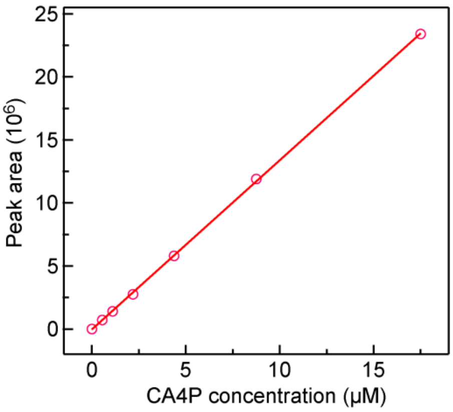

Fig. S1 Linear relationship between the peak area and the CA4P concentration in the LC–MS measurements. The red circles represent the CA4P concentration in the range of 0–17.5 μM. The equation obtained from linear fitting is $y = 1.3436 \times 10^6 x - 50700$, with the coefficient of determination being $R^2 = 0.9998$

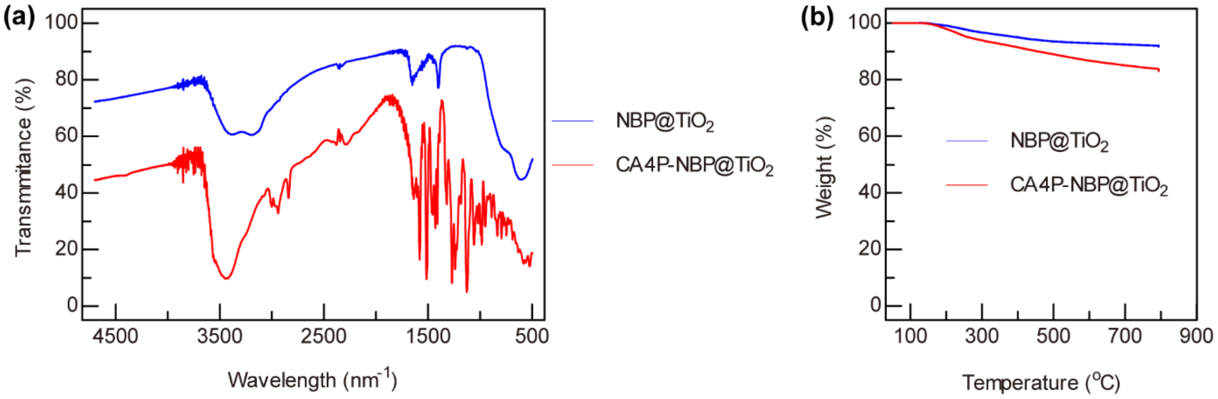

Fig. S2 a FTIR and **b** TGA analysis of $NBP@TiO_2$ and CA4P-loaded $NBP@TiO_2$ nanostructures

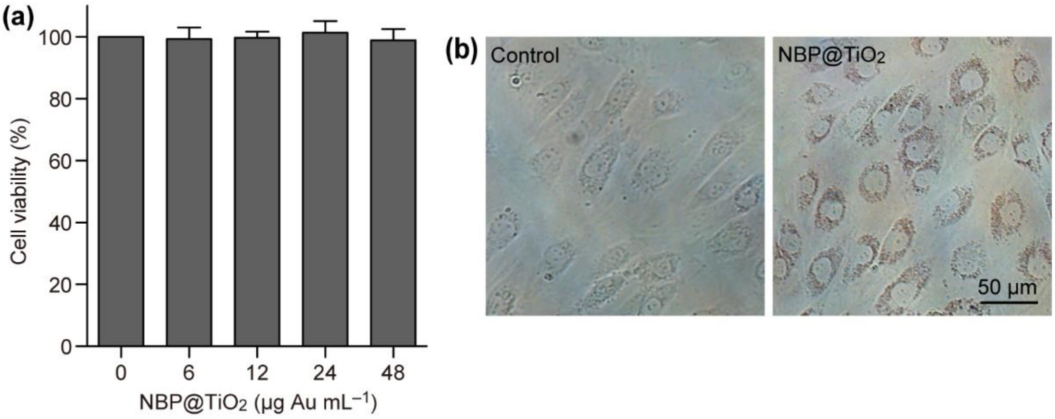

Fig. S3 Cytotoxicity and cellular uptake behavior of the $NBP@TiO_2$ nanostructures in HUVECs. **a** Cell viabilities in the presence of the nanostructures at different concentrations. HUVECs were incubated with the $NBP@TiO_2$ nanostructures (0–48 μg-Au mL^{-1}) for 48 h. The cell viability was investigated by the MTT assay. The shown data represent the mean ± SEM. **b** Optical images showing the HUVECs in the absence of the $NBP@TiO_2$ nanostructures (left) and those after the internalization of the nanostructures (right). The cells were incubated with the nanostructures (12 μg-Au mL^{-1}) for 48 h

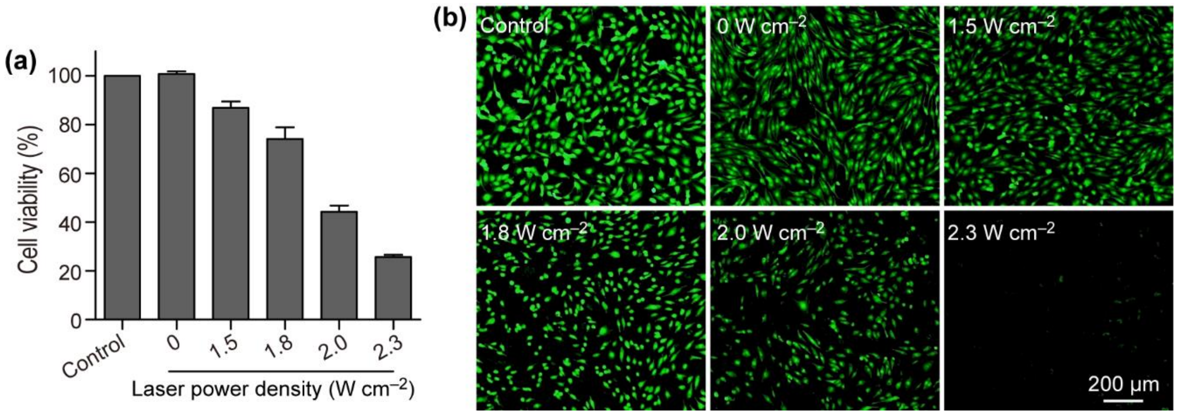

Fig. S4 PTT in HUVECs with the NBP@TiO_2 nanostructures. **a** Cell viabilities determined by the MTT assay. **b** Calcein AM staining. After incubation with the NBP@TiO_2 nanostructures (12 μg-Au mL^{-1}) for 24 h, the HUVECs were exposed to 1064-nm laser irradiation at 0–2.3 W cm^{-2} for 3 min. The assay and staining were performed after further incubation for 24 h. The shown data represent the mean ± SEM

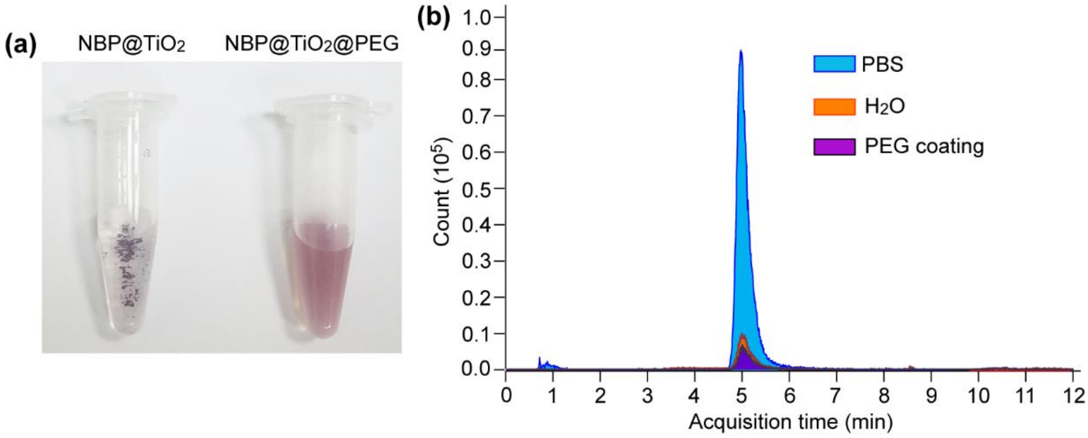

Fig. S5 Coating the NBP@TiO_2 nanostructures with the dopamine-functionalized PEG. **a** Photograph of the NBP@TiO_2 and PEG-coated NBP@TiO_2 nanostructures in the saline (0.9% NaCl) solution. The PEG coating significantly enhances the colloidal stability of the NBP@TiO_2 nanostructures. **b** LC-MS chromatograms of CA4P for the drug-loaded NBP@TiO_2 nanostructures immersed in PBS, H_2O and a dopamine-functionalized PEG solution, respectively. The PEG coating does not induce significant CA4P release from the drug-loaded NBP@TiO_2 nanostructures. The CA4P-loaded NBP@TiO_2 nanostructures (60 μg Au) were dispersed in 500 mL of PBS, H_2O and a dopamine-functionalized PEG solution (1 mg mL^{-1}) at room temperature for 2 h, respectively. The drug desorption percentage was calculated by measuring the CA4P concentration in the supernatant by LC-MS

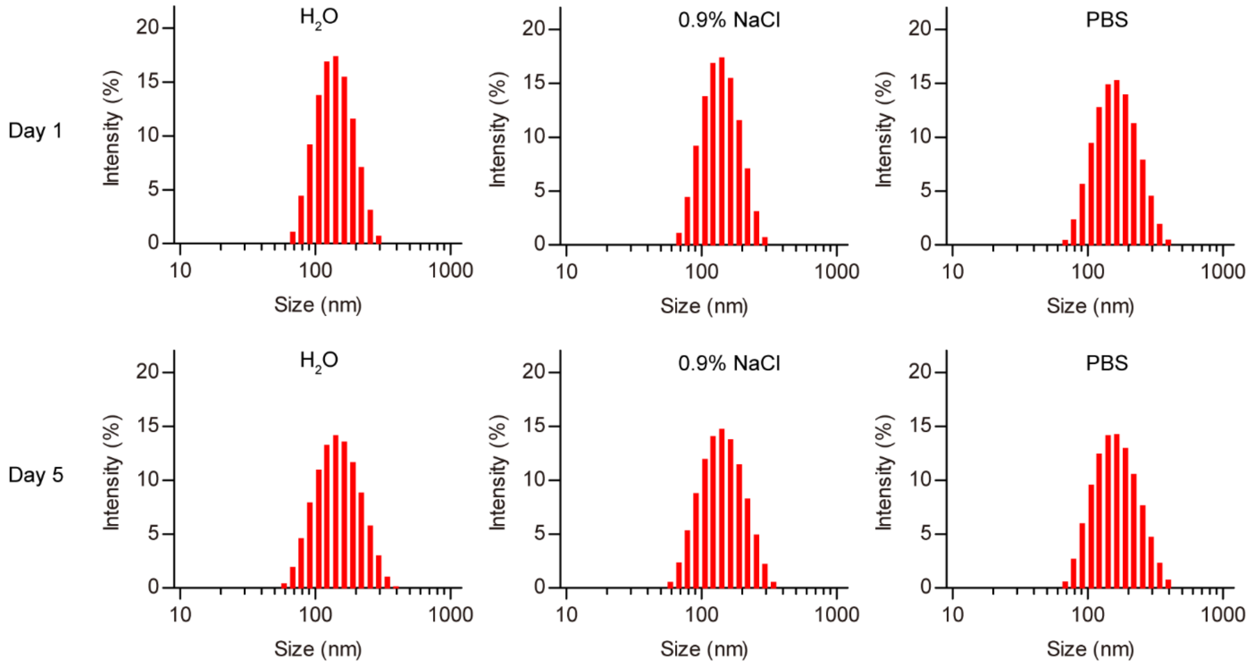

Fig. S6 Hydrodynamic size of CA4P-loaded NBP@TiO_2 nanostructures in H_2O, 0.9% NaCl or PBS after 1 or 5 day-incubation

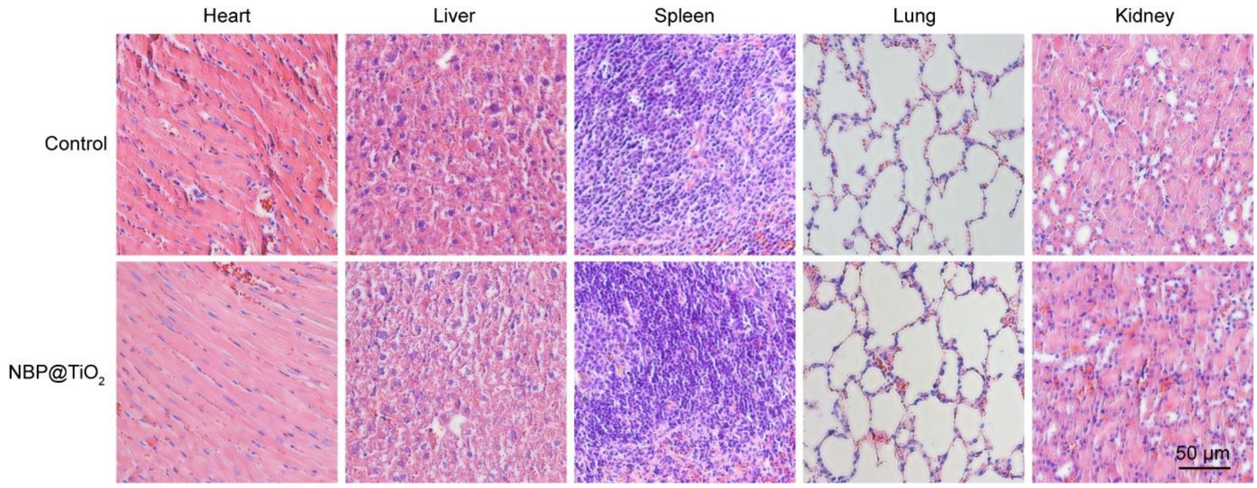

Fig. S7 H&E stained images of major organs. The mice were intravenously injected with the PEG-coated NBP@TiO_2 nanostructures (25 mg-Au kg^{-1}), and sacrificed after 20 days. Major organs were sliced and stained by H&E for histology analysis

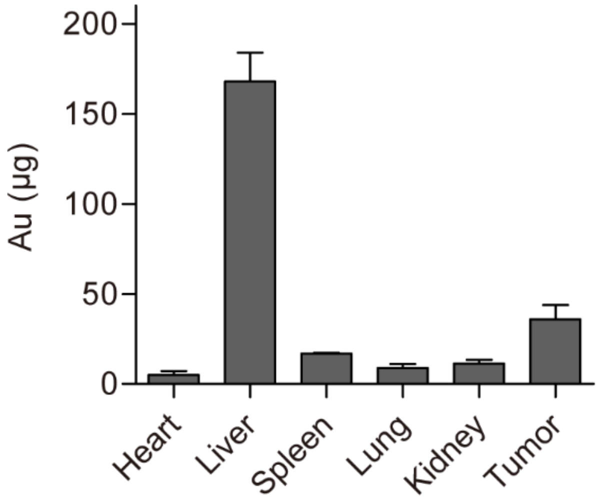

Fig. S8 Biodistribution of NBP@TiO_2 nanostructures. Au contents in different organs 24 h after single intravenous administration of the PEG-coated NBP@TiO_2 nanostructures (25 mg-Au kg^{-1}) were measured by ICP-AES. The shown data represent the mean ± SEM

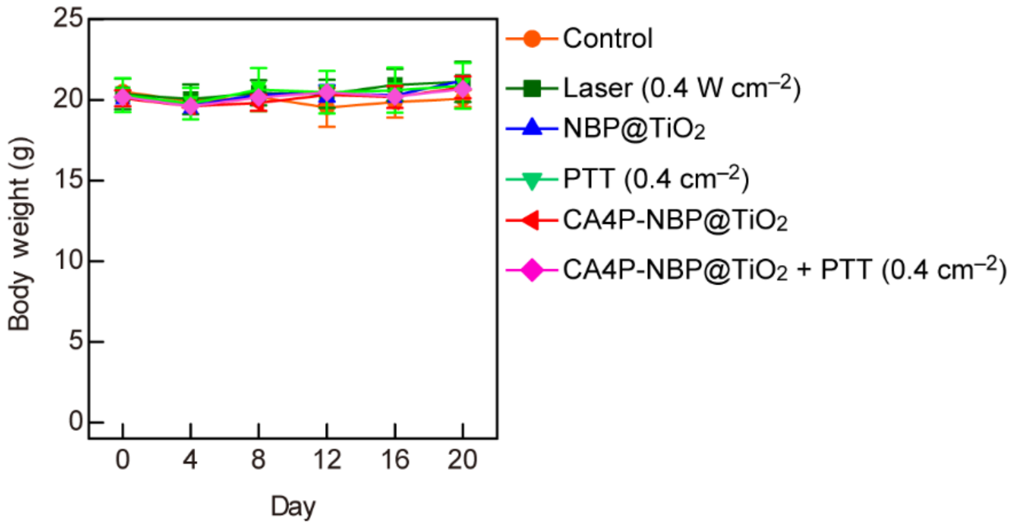

Fig. S9 The body weight of mice after various treatments. The shown data represent the mean ± SEM

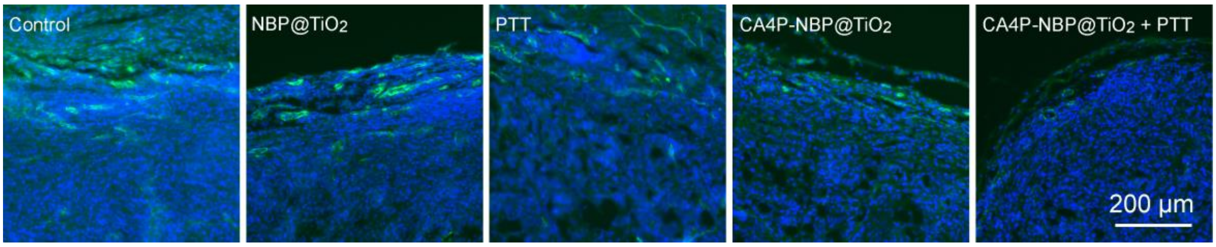

Fig. S10 Immunofluorescence images of the micro-vessels at the tumor edge with anti-CD31 (green) antibody. The nuclei were stained with Hoechst 33342 (blue)

Table S1 Complete blood count. The shown data represent the mean ± SEM

	**Control**	**NBP@TiO_2**
White blood cell ($\times10^9$ L^{-1})	5.1 ± 0.4	5.3 ± 0.4
Red blood cell ($\times10^{12}$ L^{-1})	12.5 ± 0.6	13.0 ± 0.4
Hemoglobin (g L^{-1})	187.8 ± 9.8	195.7 ± 5.5
Hematocrit (%)	52.4 ± 3.1	55. 5 ± 1.6
Mean corpuscular volume (fL)	43.7 ± 2.0	42.6 ± 0.3
Mean corpuscular hemoglobin (pg)	15.1 ± 0.2	15.0 ± 0.2
Mean corpuscular hemoglobin concentration (g L^{-1})	359.0 ± 4.1	353.0 ± 3.4
Red blood cell distribution width (%)	15.8 ± 0.2	15.5 ± 0.1
Platelets ($\times10^9$ L^{-1})	512.8 ± 64.6	538.5 ± 53.4
Plateletcrit (%)	0.32 ± 0.04	0.33 ± 0.03
Meam platelet volume (fL)	6.2 ± 0.1	6.2 ± 0.1
Platelet distributionwidth (%)	12.3 ± 0.2	12.0 ± 0.7
Lymphocyte ($\times10^9$ L^{-1})	0.82 ± 0.27	0.86 ± 0.19
Monocytes ($\times10^9$ L^{-1})	0.23 ± 0.10	0.28 ± 0.13
Neutrophil ($\times10^9$ L^{-1})	3.7 ± 0.6	3.8 ± 0.4
Eosinophils ($\times10^9$ L^{-1})	0.10 ± 0.05	0.1 ± 0.05
Basophil ($\times10^9$ L^{-1})	0.28 ± 0.28	0.29 ± 0.28
